# Ifosfamide-based combination chemotherapy in advanced soft-tissue sarcoma: a practice guideline

**DOI:** 10.3747/co.2007.130

**Published:** 2007-08

**Authors:** S. Verma, J. Younus, D. Stys–Norman, A.E Haynes, M. Blackstein

**Affiliations:** * Division of Medical Oncology, Ottawa Hospital, Ottawa, Ontario; † London Regional Cancer Centre, London, Ontario.; ‡ Cancer Care Ontario, Program in Evidence-Based Care, McMaster University, Hamilton, Ontario.; § Mount Sinai Hospital, Toronto, Ontario

**Keywords:** Ifosfamide, chemotherapy, soft tissue, sarcoma, practice guideline

## Abstract

**Questions:**

In adult patients with inoperable locally advanced or metastatic soft-tissue sarcoma, do combination chemotherapy regimens containing ifosfamide have an advantage in terms of response rate, time to progression, or survival, as compared with similar regimens without ifosfamide when used as first-line therapy?

What are the adverse effects and effects on quality of life of ifosfamide-containing combination chemotherapy as compared with similar regimens without ifosfamide?

**Perspectives:**

The prognosis for patients with inoperable or meta-static soft-tissue sarcoma (sts) remains grim. Although the surgical resection of pulmonary metastases may be curative in 15%–30% of patients with isolated slow-growing metastases, most patients receive chemotherapy for palliative purposes. Ifosfamide has documented activity in patients who have received prior treatment with, or who have progressed on, doxorubicin. A number of studies have suggested a schedule and a dose–response relationship for ifosfamide in metastatic sts. Ifosfamide has also been assessed in combination with other drugs such as doxorubicin and dacarbazine (dtic); results of such studies have led some authors to suggest that polychemotherapy using “appropriate doses” of ifosfamide and doxorubicin may represent the “most effective systemic treatment” in this population. Given the limited effective therapeutic options available for patients with metastatic sts, the Sarcoma Disease Site Group (dsg) felt that a need existed to more specifically evaluate the potential benefits of ifosfamide-containing combination chemotherapy in that setting. The Sarcoma dsg developed an evidence-based series report through systematic review, evidence synthesis, and input from practitioners across Ontario.

**Outcomes:**

Outcomes of interest included survival, response rate, adverse events, and quality of life.

**Methodology:**

A systematic review and meta-analysis served as the evidentiary base for this clinical practice guideline. The report was reviewed and approved by the Sarcoma dsg, which comprises medical oncologists, radiation oncologists, surgeons, methodologists, and patient representatives. The results of an external review by Ontario practitioners, obtained through a mailed survey, were incorporated into this report. Final approval of the evidence-based series report was obtained from the Report Approval Panel of Cancer Care Ontario’s Program in Evidence-Based Care (pebc).

**Results:**

The current practice guideline reflects a combination of the draft recommendations (based on the evidence identified in a systematic review and meta-analysis) and the external feedback from Ontario practitioners and the pebc’s Report Approval Panel.

**Practice Guideline:**

In patients with metastatic sts, the addition of ifosfamide to standard first-line doxorubicin-containing regimens is not recommended over single-agent doxorubicin.

However, in patients with symptomatic, locally advanced, or inoperable sts, in whom tumour response might potentially result in reduced symptomatology or render a tumour resectable, use of ifosfamide in combination with doxorubicin is reasonable.

**Qualifying Statement:**

In combination with a doxorubicin-containing regimen, the dose of ifosfamide should not exceed 7.5 g/m^2^, given as either a split bolus or a continuous infusion.

## 1. QUESTIONS

In adult patients with inoperable locally advanced or metastatic soft-tissue sarcoma (sts), do combination chemotherapy regimens containing ifosfamide have an advantage in terms of response rate, time to progression, or survival, as compared with similar regimens without ifosfamide when used as first-line therapy?

What are the adverse effects and effects on quality of life of ifosfamide-containing combination chemotherapy as compared with similar regimens without ifosfamide?

## 2. CHOICE OF TOPIC AND RATIONALE

The prognosis for patients with inoperable or meta-static sts remains grim. Although surgical resection of pulmonary metastases may be curative in 15%–30% of patients with isolated, slow-growing metastases 1,2, most patients receive chemotherapy for palliative purposes.

Ifosfamide has documented activity in patients who have received prior treatment with, or who have progressed on, doxorubicin 3–6.A number of studies have suggested a schedule and dose–response relationship for ifosfamide in metastatic sts 6–8. The drug has also been assessed in combination with other drugs such as doxorubicin and dacarbazine (dtic) 6,9,10. The results of such studies have led some authors to suggest that polychemotherapy with “appropriate doses” of ifosfamide and doxorubicin may represent the “most effective systemic treatment” in this population.

Given the limited effective therapeutic options available to patients with metastatic sts, the Sarcoma dsg felt that a need existed to more specifically evaluate the potential benefits of ifosfamide-containing combination chemotherapy in that setting.

## 3. METHODS

### 3.1 Guideline Development

The present practice guideline was developed by the Sarcoma Disease Site Group (dsg) of Cancer Care Ontario’s Program in Evidence-based Care (pebc), using the methods of the practice guidelines development cycle 11. This practice guideline is a convenient and up-to-date source of the best available evidence on ifosfamide-based combination chemotherapy for patients with inoperable locally advanced or metastatic sts. The body of evidence in this report is composed primarily of data from mature randomized controlled trials (rcts). That evidence forms the basis of a clinical practice guideline developed by the Sarcoma dsg. The systematic review (which is under consideration for publication elsewhere) and the companion practice guideline are intended to promote evidence-based practice in Ontario, Canada. The evidence was selected and reviewed by one member of the Sarcoma dsg and by methodologists. Members of the Sarcoma dsg disclosed information on potential conflicts of interest. No conflicts were declared. The pebc is editorially independent of Cancer Care Ontario and the Ontario Ministry of Health and Long-Term Care.

External review is obtained for all practice guideline reports through a mailed survey of Ontario practitioners. The survey consists of items that address the quality of the draft practice guideline report and recommendations, and that ask whether the recommendations should serve as a practice guideline. Final approval of the practice guideline report is obtained from the pebc’s Report Approval Panel (rap).

### 3.2 Literature Search Strategy

The medline (1966 to July 2005), embase (1980 to July 2005), and Cochrane Library (2004, Issue 3) databases were systematically searched for eligible randomized controlled phase ii and iii trials, practice guidelines, systematic reviews, and meta-analyses. In addition, conference proceedings of the American Society of Clinical Oncology (1997 to Spring 2005) were searched for abstracts of relevant trials.

## 4. LITERATURE SEARCH RESULTS

The literature search identified three randomized trials phase iii^9^,^10^,^12^ and twenty-three single-arm phase trials ii^6^,^13^–^34^ that met the inclusion criteria for this systematic review of the evidence.

All three rcts 9,10,12 and sixteen of the phase ii trials 6,14–28 used ifosfamide with an anthracycline (doxorubicin or epirubicin). The remaining seven phase studies ii^13^,^29^–^34^ used ifosfamide in combinations that did not include an anthracycline. Two of s the rct 10,12 reported that the addition of ifosfamide to an anthracycline regimen (either doxorubicin, or doxorubicin and dtic) significantly improved the response rate (34%, *p* = 0.03, and 32%, *p* < 0.005, respectively). The by Antman et al. rct^12^ also reported a significant improvement in overall survival (*p* = 0.04) for patients who received doxorubicin and dtic as compared with patients who received maid (mesna, doxorubicin, ifosfamide, and dtic). The reason for that finding cannot be discerned from the trial, but histologic differences in the trial population could possibly have resulted in subtle imbalances in the treatment arms.

All three rcts reported higher rates of adverse events in the regimens that contained ifosfamide. Two of the trials reported that grades 3 and 4 adverse events were much higher in the ifosfamide arm 10,12. Both trials reported a total of 11 toxic deaths, plus increased hematologic toxicity [myelosuppression (80%) and leucopenia (86%)].

Although the dsg identified and referenced a number of phase ii trials that conformed to the inclusion criteria for the present guideline, the results of those trials were non-contributory to the final guideline recommendations. The original Sarcoma dsg report (found at www.cancercare.on.ca/) includes the complete phase ii information.

## 5. DSG CONSENSUS PROCESS

The draft guideline was circulated to the Sarcoma dsg for review and discussion. The group agreed that, although the available evidence indicates that the addition of ifosfamide may improve tumour response, that improvement does not translate into a survival benefit. The evidence also indicates that treatment-related toxicities are clearly increased with the addition of ifosfamide to doxorubicin-containing regimens.

## 6. PRACTITIONER FEEDBACK

The Sarcoma dsg circulated the draft clinical practice guideline and systematic review to practitioners in Ontario for review and feedback.

### 6.1 Methods

Feedback was obtained through a mailed survey of 74 Ontario practitioners, including medical oncologists, radiation oncologists, and surgeons. The survey consisted of items evaluating the methods, results, and interpretive summary used to inform the draft recommendations and asking whether the draft recommendations should be approved as a practice guideline. Written comments were invited. The survey was mailed February 22, 2006. Follow-up reminders were sent at 2 weeks (post card) and 4 weeks (complete package mailed again). The Sarcoma dsg reviewed the results of the survey.

### 6.2 Results

From among 74 surveys mailed, 29 responses were received (39% response rate). Responses included returned completed surveys, plus telephone, fax, and e-mail responses.

Of the practitioners who responded, 9 indicated that the report was relevant to their clinical practice, and they completed the survey. One practitioner was unsure whether the guideline was relevant to personal practice, and so that practitioner’s comments were not included in the results. Another indicated that the topic was relevant, but did not complete the questionnaire because of concerns that direct contact with patients was not part of current personal work. Of the respondents surveyed, most agreed (87.5%) that a need for a guideline on this topic existed, that the recommendations in the report were clear, and that the draft report should be approved as a practice guideline. All practitioners surveyed agreed with the recommendations as stated in the draft.

### 6.3 Summary of Written Comments and Modifications/Actions

Of the 9 survey respondents, 1 provided suggestions for future document development and content. Those suggestions were noted at the pebc. One practitioner noted an error regarding the presentation of study results in the Discussion section. The error was corrected in the report.

## 7. REVIEW AND APPROVAL BY THE PEBC REPORT APPROVAL PANEL

The final evidence-based series report was also reviewed and approved by the pebc rap, which consists of two members, including an oncologist with expertise in clinical and methodologic issues. Key issues raised by the rap were that the inclusion of the word “routine” in the recommendation created ambiguity in light of the compelling evidence demonstrating lack of benefit, and that a rationale for using response as an important and policy-determining outcome was required, as was a rationale for including phase ii studies, given the availability of three RCTs.

In response, the dsg removed the word “routine”; noted that, given the limited treatment options, response is an important outcome in this patient population; and noted that the inclusion of phase ii studies reflects an approach previously used (at the time the report was initially started) of including rcts and phase ii studies alike.

## 8. PRACTICE GUIDELINE

The current practice guideline integrates the draft practice guideline with feedback gathered by external review. This report has been approved by the Sarcoma dsg and the pebc rap.

### 8.1 Recommendation

In patients with metastatic sts, the addition of ifosfamide to standard first-line doxorubicin-containing regimens is not recommended over single-agent doxorubicin. However, in patients with symptomatic locally advanced or inoperable STS, in whom tumour response might potentially result in reduced symptomatology or render a tumour resectable, it is reasonable to use ifosfamide in combination with doxorubicin.

### 8.2 Qualifying Statements

In combination with a doxorubicin-containing regimen, the dose of ifosfamide should not exceed 7.5 g/m^2^, given as either a split bolus or a continuous infusion.

## 9. PRACTICE GUIDELINE DATE

Completed April 2006. Practice guidelines developed by the pebc are reviewed and updated regularly. Please visit the Cancer Care Ontario Web site (www.cancercare.on.ca/) for the full evidence-based series report and subsequent updates.
